# Neuroprotective Effects and Hepatorenal Toxicity of Angong Niuhuang Wan Against Ischemia–Reperfusion Brain Injury in Rats

**DOI:** 10.3389/fphar.2019.00593

**Published:** 2019-05-29

**Authors:** Bun Tsoi, Xingmiao Chen, Chong Gao, Songlin Wang, Sau Chu Yuen, Depo Yang, Jiangang Shen

**Affiliations:** ^1^School of Chinese Medicine, Li Ka Shing Faculty of Medicine, The University of Hong Kong, Hong Kong, Hong Kong; ^2^School of Pharmaceutical Science, Sun Yat-sen University, Guangzhou, China

**Keywords:** Angong Niuhuang Wan, cerebral ischemia–reperfusion injury, blood–brain barrier, heavy metal, safety

## Abstract

Angong Niuhuang Wan (AGNHW) is a classic prescription in traditional Chinese medicine (TCM) used for stroke treatment, but its efficacies remain to be confirmed. With its arsenic- and mercury-containing materials, the application of AGNHW raises great safety concerns. Herein, we aim to explore the neuropharmacological effects against cerebral ischemia–reperfusion injury and evaluate the toxicological effects of AGNHW for better use. Male SD rats were subjected to 2 h of middle cerebral artery occlusion (MCAO) and following 22 h of reperfusion. AGNHW (257 mg/kg, 1× AGNHW) were orally administered for pharmacological effects and 257, 514, and 1,028 mg/kg (equivalent to 1×, 2×, 4× AGNHW) were used for the toxicological study. The results revealed that AGNHW treatment reduced the infarct size and protected the blood–brain barrier (BBB) integrity in the MCAO rat ischemic stroke model. AGNHW treatment up-regulated bcl-2 expression and down-regulated the expressions of Bax, p47_phox_, inducible nitric oxide synthase (iNOS), and 3-nitrotyrosine (3-NT), and inhibited the expressions and activities of matrix metalloproteinase-2 (MMP-2), MMP-9, and reserved tight junction protein zonula occludens-1 (ZO-1) and claudin-5 in the ischemic brains. These results indicated that the neuroprotective mechanisms of AGNHW could be associated with its antioxidant properties by inhibiting oxidative/nitrative stress-mediated MMP activation and protecting tight junction proteins in the ischemic brains. Administration of 1× AGNHW for 7 days would not induce the accumulation of mercury in blood, liver, and kidney at day 14. Administration of 2× AGNHW and 4× AGNHW for 7 days increased the level of mercury in the kidney. For arsenic level, administration of 1× AGNHW for 7 days would increase the level of arsenic in the liver and blood without increase of arsenic in the kidney at day 14. Administration of 2× AGNHW and 4× AGNHW for 7 days would further increase the level of arsenic in the liver and blood. There is no influence on body weight, organ index, histological structures, and renal and liver functions. These results suggest that short-term treatment of AGNHW within 1 week should be safe and has neuroprotective effects against cerebral ischemia–reperfusion injury.

## Introduction

Stroke is the second cause of death (World Health Organization, 2018) and the leading cause of disability in human diseases (Feigin et al., 2017). Among them, ischemic stroke accounts for around 80% of all stroke cases (Rai et al., 2016). Although advanced computed tomography and magnetic resonance imaging can facilitate the diagnosis of the type of stroke and the area of the brain affected by stroke, millions of neurons will still be destroyed every minute (Saver, 2006). To date, intravenous thrombolysis is the only FDA-approved treatment for ischemic stroke but carries restrictive therapeutic window, which is within 4.5 h of cerebral ischemia (Catanese et al., 2017). It is necessary to develop new therapeutic approaches and drugs to lengthen the therapeutic window and ameliorate the consequences of cerebral ischemia–reperfusion injury.

Blood–brain barrier (BBB) disruption is a critical pathological process in cerebral ischemia–reperfusion injury. Matrix metalloproteinases (MMPs) are responsible for degradation of extracellular matrix around cerebral blood vessels and neurons, causing BBB opening, hemorrhage transformation (HT), and ischemic brain injury (Lakhan et al., 2013). Serum MMPs are important biomarkers for predicting BBB disruption and HT in ischemic stroke patients. Clinical trials indicated that early plasma MMP-9 level was correlated with infarct severity and BBB damage, which could be used for predicting hemorrhagic complications in stroke patients (Castellanos et al., 2007). Recanalization with delayed thrombolysis could induce oxidative stress, mediate MMP activation, and lead to BBB disruption in cerebral ischemia–reperfusion injury (Gasche et al., 2001; Jian Liu and Rosenberg, 2005). During cerebral ischemia–reperfusion injury, large quantities of free radicals are produced in the ischemic brains, including reactive oxygen species (ROS) and reactive nitrogen species (RNS). Free radicals could mediate leukocyte adhesion, MMP activations, and BBB disruption (Chen et al., 2013; Chen et al., 2018). Superoxide (O_2_^−^) and nitric oxide (NO) can rapidly react with each other to form peroxynitrite (ONOO^−^) with diffusion limit (*k*_2_ = 4.7 × 10^9^ M^−1^ s^−1^). Peroxynitrite can easily penetrate the membrane and have far more activity than its precursors. As a nitrative product of ONOO^−^, 3-nitrotyrosine (3-NT) was increased in the ischemic penumbral cortex of stroke animal and in plasma of stroke patients (Suzuki et al., 2002; Bas et al., 2012). Peroxynitrite decomposition catalyst (PDC) can neutralize ONOO^−^ to form non-toxic nitrates. PDC was found to attenuate infarction volume, brain edema, and neurological deficits in focal ischemic rat brains (Thiyagarajan et al., 2004). Our recent studies indicated that PDC could prevent HT and improve neurological outcome in ischemic rat brains with delayed t-PA treatment via inhibiting peroxynitrite-mediated MMP activation (Chen et al., 2015). Therefore, targeting RNS-mediated MMP activation could be an important therapeutic strategy for protecting BBB integrity, improving therapeutic outcome and reducing cerebral ischemia–reperfusion injury during thrombolytic treatment.

Traditional Chinese Medicine (TCM) has a long history, and there were many famous TCM formulas for stroke treatment. Angong Niuhuang Wan (AGNHW) is one of the representative prescriptions for the treatments of traumatic brain injury and stroke (Guo et al., 2014). AGNHW consists of 11 herbal and mineral medicines, including *Calculus bovis* (5.56%), powder of* Cornu bubali* (11.11%), *Moschus* (1.39%), *Margarita* (2.78%), *Cinnabaris* (5.56%), *Realgar* (5.56%), *Coptis chinensis* Franch. (5.56%), *Scutellaria baicalensis* Georgi (5.56%), *Gardenia jasminoides* J. Ellis (5.56%), *Curcuma aromatica* Salisb. (5.56%), and *Borneolum synthcticum* (1.39%). With the detoxification, resuscitation, and anticonvulsant properties, the formula is specifically used for the clinical syndrome patterns with high fever, unconsciousness, convulsion, several headaches, dizziness, nausea and vomiting, difficulty in moving, hemiplegic paralysis, language disorders, neck rigidity, epilepsy, and constipation in classic TCM practice. Thus, AGNHW was named “one of the three treasures” and often used as first-aid drug for emergency cases in ancient time. Clinical studies had shown that AGNHW could improve neurological impairment in ischemic stroke patients (Xing et al., 2005; Luo, 2009). Add-on treatment of AGNHW with conventional medicine showed the beneficial effects over conventional medicine alone in stroke patients (Wang and Wen, 2010; Wu et al., 2012). AGNHW has synergistic effects with conventional medicine for reducing the mortality rates in acute cerebral hemorrhage patients (Zhang and Li, 2013). Experimentally, AGNHW revealed its neuroprotective effects via its antioxidant and anti-inflammatory properties in rat models of cerebral ischemia injury (Wang et al., 2014). However, the neuroprotective mechanisms of AGNHW are largely unclear.

On the other hand, there are major safety concerns about the heavy metal components in the formula named cinnabar (mercuric sulfide, >96% HgS) and realgar (arsenic sulfide, >90% As_4_S_4_), respectively. Whether the metal materials of AGNHW are toxic raises great concerns because AGNHW is widely used in TCM practice. In the present study, we tested the hypothesis that AGNHW could protect the BBB integrity and decrease infarction size and neurological deficits in cerebral ischemia–reperfusion injury via inhibiting ROS/RNS-mediated MMP activation and protecting tight junction (TJ) proteins. Moreover, we systematically evaluated the potential effects of AGNHW on the liver and renal toxicity and the accumulation of arsenic and mercury in the blood, liver, and kidney.

## Materials and Methods

### Animals

Male Sprague–Dawley (SD) rats weighing 220–240 and 260–280 g were obtained from Laboratory Animal Unit, The University of Hong Kong. This study was carried out in accordance with the recommendations of *Guidelines for the Use of Experimental Animals*, the Committee on the Use of Live Animal in Teaching and Research, The University of Hong Kong. The protocol was approved by the Committee on the Use of Live Animal in Teaching and Research, The University of Hong Kong.

### AGNHW Preparation and Quantitative Analysis

AGNHW was kindly provided by Beijing Tong Ren Tang Chinese Medicine Co. Ltd. In order to mimic human dosage (one 3-g pill per day), AGNHW was suspended in normal saline and given to animals at a concentration of 257 mg/kg (equivalent to 1× human daily dose, 1× AGNHW). For toxicological studies, AGNHW was prepared in three concentrations at 257 mg/kg (equivalent to 1× human daily dose, 1× AGNHW), 514 mg/kg (equivalent to 2× human daily dose, 2× AGNHW), and 1,028 mg/kg (equivalent to 4× human daily dose, 4× AGNHW).

For quantitative analysis of AGNHW, baicalin, berberine, and geniposide (Chengdu Herbpurify Co., Ltd., China) were used as standards of quality control. In brief, AGNHW was shredded, ground, and mixed with diatomaceous earth (Sigma-Aldrich, MO, USA) in 1:1 ratio. Accurately weighed 1.0 g of AGNHW mixture was put into a conical flask and 25 ml of 75% ethanol (VWR Chemicals, PA, USA) was added to proceed to sonification for 30 min. Baicalin, berberine, and geniposide were accurately weighed and mixed with methanol (VWR Chemicals, PA, USA) in a series of concentration. All solutions were then filtered through a 0.22-μm filter before injection into the high-performance liquid chromatography (HPLC) system. HPLC analysis for baicalin, berberine, and geniposide was performed by Thermo Scientific UltiMate 3000 HPLC system, equipped with a diode-array detector (ThermoFisher Scientific, MA, USA). Separation of samples was performed by an ACE 5 AQ-C_18_ column (5 μm, 4.6 × 250 mm, Advanced Chromatography Technologies Ltd.), with a gradient mobile phase system using acetonitrile (ACN)–0.2% phosphoric acid (VWR Chemicals, PA, USA). The gradient profile was started with 20% ACN and increased to 23% at 10 min. The ACN then further increased to 25% at 20 min, to 50% at 30 min, and finally to 90% at 35 min. The column temperature was maintained at 30°C. The flow rate was set at 1.0 ml/min and the injection volume was 10 μl. The analysis was detected at 254 nm. All solvents and chemicals used were of analytical grade.

### Middle Cerebral Artery Occlusion Model

Male SD rats weighing 260–280 g were used. Rats were randomly divided into the groups of sham, middle cerebral artery occlusion (MCAO) model, and AGNHW (257 mg/kg, 1× AGNHW). The cerebral ischemia–reperfusion MCAO model was conducted according to the previous protocol (Feng et al., 2018b). Briefly, MCAO rats were anesthetized with 4% isoflurane (Abbott, IL, USA) and maintained with 1.5% isoflurane via inhalation. The common carotid artery (CCA), external carotid artery (ECA), and internal carotid artery (ICA) was exposed and ligated on the left side. A 0.36-mm monofilament suture with a silicon-coated tip (L3600, Jialing Co. Ltd., China) was inserted into the ECA and advanced through the ICA to the ostium to occlude the middle cerebral artery. Sham control rats were subjected to similar surgical operation without occlusion. After 2 h of ischemia, the monofilament suture was withdrawn to permit reperfusion. Meanwhile, the aqueous solution of AGNHW or saline was orally administered to the animal immediately before reperfusion. The reperfusion process continued for 22 h. Rats were allowed to free access of food and water after recovery from anesthesia.

### Neurological Deficit Measurement

The Modified Neurological Severity Score (mNSS) test was adopted to evaluate the animal neurological deficit right before being sacrificed for further experiments (Zhang et al., 2002). Neurological functions, including motor, sensory, reflex, and balance, were graded on a series of scales from 0 to 18 (normal score, 0; maximal deficit score, 18).

### Infarct Size Measurement

All animals were transcardially perfused with PBS under anesthesia to collect brain tissues for further experiments. The isolated brains were sectioned into 2-mm slices with a rat brain matrix (RWD Life Science, Shenzhen, China) and placed in 2% 2,3,5-triphenyltetrazoliumchloride (TTC, Sigma-Aldrich, MO, USA) solution for 20 min in the dark. Infarction size was quantified by measuring the white infarcted area and reddish-purple non-infarcted area using Image J software. Calculation of infarct size was according to the following formula: infarct size percentage = [the size of right hemisphere − red size of left hemisphere)/2× right hemisphere size] × 100%.

### Evaluation of Blood–Brain Barrier Integrity

A 2% solution of Evans Blue (EB, Sigma-Aldrich, MO, USA) in normal saline (4 ml/kg of body weight) was injected into the tail vein 3 h before sacrificed. Afterwards, the animals were perfused with PBS to collect brain tissue. The brain tissue was sliced into 2-mm slices and then divided into left and right hemispheres. The left hemisphere was homogenized in PBS and then mixed with twice the volume of 50% trichloroacetic acid (Sigma-Aldrich, MO, USA). The mixture was immediately centrifuged (20 min, 20,000×*g*, 4°C). Supernatants were collected and the EB content in the supernatant was measured by a spectrophotometer (Model 680, Bio-Rad) at 620 nm and quantified according to a standard curve. The results are presented as (μg of Evans Blue stain)/(g of tissue).

### Isolation of Brain Microvessels

Isolation of brain microvessels was performed according to our previous report with slight modifications (Gu et al., 2012). PBS-perfused brain tissue was divided into left and right hemispheres. The big vessels on the surface of the left hemisphere were removed by filter paper and then homogenized by PBS. After discarding the supernatant, the homogenized brain tissue was mixed and digested with 20% BSA-DMEM solution (Invitrogen, CA, USA). Microvessels were collected by centrifuge at 1,000×*g*, 4°C for 20 min. The precipitates (microvessels) were washed with PBS twice and then lysed with RIPA buffer (Sigma-Aldrich, MO, USA) for further analysis.

### Western Blot

Brain tissues and microvessels were lysed according to standard Western blot protocol. Equal amounts of proteins were separated by SDS-polyacrylamide gel electrophoresis, transferred onto a 0.45-μm polyvinylidene fluoride membrane, and blocked with 5% BSA blocking buffer. The membrane is then incubated with antibodies for Bax (CST), bcl-2 (CST), NADPH oxidase (p47_phox_, Santa Cruz), iNOS (Abcam), eNOS (Invitrogen), 3-NT (Abcam), MMP-9 (Santa Cruz), MMP-2 (Santa Cruz), claudin-5 (Invitrogen), and ZO-1 (Invitrogen) in blocking buffer (all in dilutions of 1:1,000). Bands were detected by enhanced chemiluminescence staining (GE Healthcare, IL, USA) and visualized by BIO-RAD ChemiDoc™ XRS+ System (USA).

### Gelatin Zymography

MMP activity was determined by gelatin zymography as previously described (Chen et al., 2015). Equal amounts of proteins were separated by SDS-polyacrylamide gel containing 1 mg/ml gelatin (Sigma-Aldrich, MO, USA). The SDS gel was washed with 2.5% Triton X-100 and then incubated with developing buffer at 37°C for 72 h. After incubation, the gel was stained with Coomassie blue (Bio-Rad, CA, USA) for band visualization.

### Immunofluorescent Staining

After being perfused with PBS, the rat brains were fixed in 4% PFA and then immersed in 30% sucrose solution for dehydration. The fixed brains were mounted with O.C.T. compound (Leica, Germany) and the coronal brain sections (35 μm thick) were cut using a microtome-cryostat (Leica, Germany) at −20°C. The frozen sections were incubated with primary antibodies against Claudin-5 and ZO-1 (1:400 in goat serum) overnight at 4°C, followed with incubation of fluorescent-conjugated secondary antibodies (Alexa Flour 488 and 568, Invitrogen, 1:400). Finally, all sections were counterstained with 4′,6-diamidino-2-phenylindole (DAPI, Sigma-Aldrich, MO, USA) and cover-slipped. Immunofluorescent images were captured using a confocal laser scanning microscope, LSM 780 (Carl Zeiss, Germany).

### Toxicology

Male SD rats weighing 220–240 g were used. Rats were randomly divided into control and different concentrations of AGNHW groups. All animals receive oral administration of saline or AGNHW (257, 514, and 1,028 mg/kg) once per day for 7 consecutive days. From the 8th day onwards, all animals were allowed to recover from drug administration until the 14th day. On the 2nd, 8th, and 14th day, blood samples were collected from the tail vein or by cardiac puncture and mixed with heparin sodium (50 U/ml blood, Millipore, MA, USA) under anesthesia. Animal body weight was recorded daily, and different organs were weighed and collected on the 14th day for further experiments. Organ index was calculated as (mg of organ weight)/(animal body weight (g).

### Liver and Kidney Function Tests

Blood samples were centrifuged at 1,000×*g* for 15 min at 4°C by refrigerated centrifuge to obtain the plasma. The levels of ALT, AST, BUN, and Cr in the plasma were measured using commercial kits (Nanjing Jiancheng Bioengineering Institute).

### Histological Analysis

Small sections of fresh liver and kidney tissues were fixed in 4% polyformaldehyde, followed by dehydration and embedded in paraffin wax. Tissue sections (4 μm) were stained with hematoxylin and eosin (H&E, Sigma-Aldrich, MO, USA) for examination of liver and kidney damage under optical microscopy (Olympus, NY, USA).

### Inductive Coupled Plasma–Mass Spectrometry

Whole blood (0.5 ml) and organ (∼0.2 g) samples were digested by concentrated nitric acid (1 ml, 67% v/v, VWR Chemicals, PA, USA) in an 85°C hot water bath for 3 h. During acid digestion, hydrogen peroxide (1 ml, Merck, Germany) was carefully added to each sample to facilitate oxidation. After complete digestion, all samples were transferred to a volumetric flask and diluted to a final volume of 20 ml by using double deionized water. Analyses of arsenic (As) and mercury (Hg) were conducted by inductive coupled plasma–mass spectrometry (ICP-MS) (Thermo Scientific iCAP-Q ICP-MS). The quality of data was checked by the analysis of recovery rate using standard reference materials (bovine liver, NIST SRM 1577c, Sigma-Aldrich, MO, USA).

### Statistical Analysis

Values are presented as means ± S.E.M. Statistical significance was determined by one-way ANOVA followed by Dunnett’s multiple-comparison test for multiple comparisons. The numbers of rats used are described in the corresponding figure legends. All experiments were repeated three or more times. All data were analyzed using GraphPad Prism (Version 6.0, GraphPad Software Inc., CA, USA). Two-sided *p* < 0.05 was considered as statistically significant.

## Results

### Quality Control Study on Angong Niuhuang Wan

**Figure 1:**
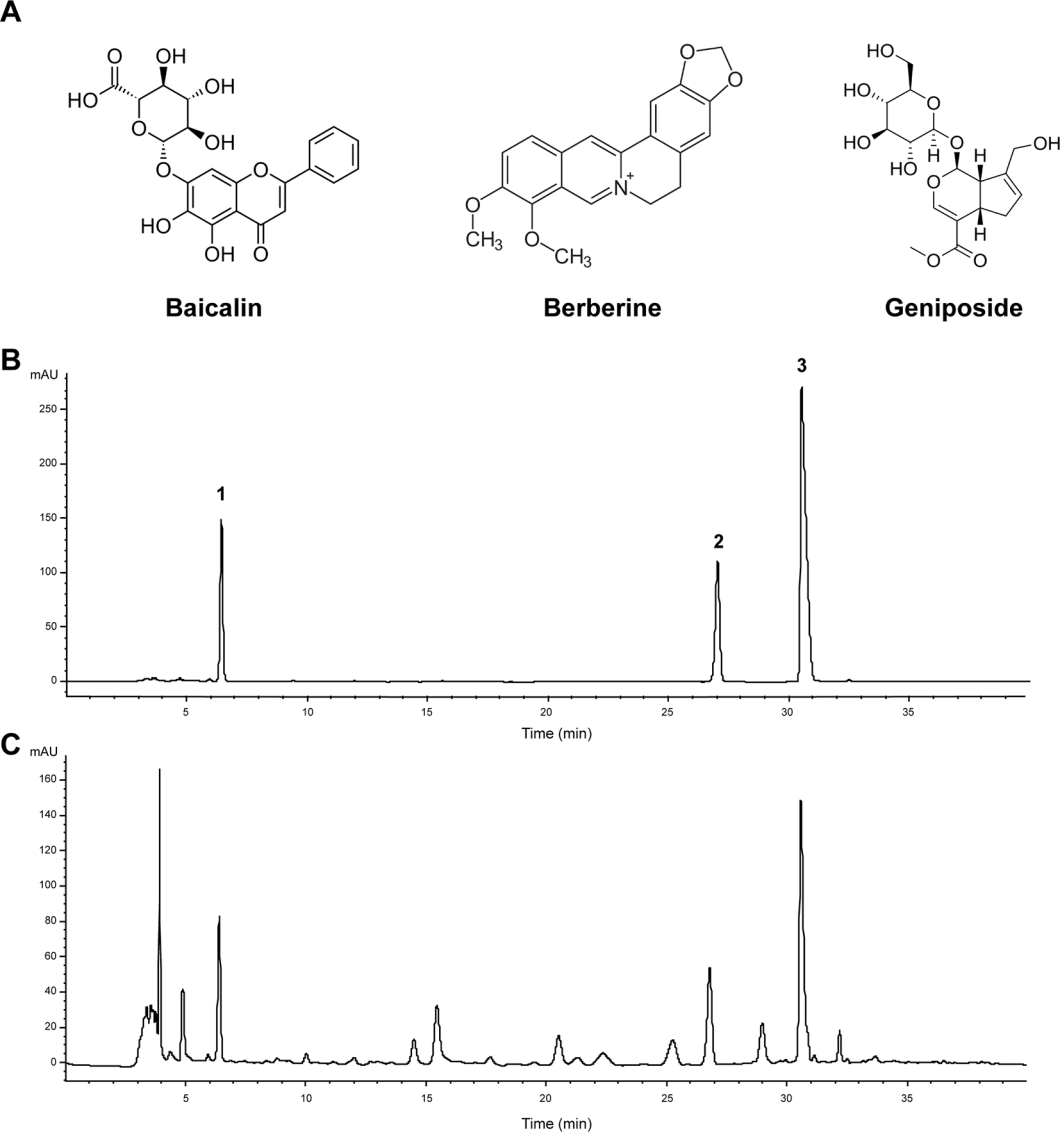
Quantitative analysis of Angong Niuhuang Wan (AGNHW). **(A)** Chemical structures of baicalin, berberine, and geniposide. **(B)** HPLC profiles for mixed standards of baicalin, berberine, and geniposide. Peak 1, geniposide; Peak 2, baicalin; Peak 3, berberine. **(C)** HPLC profile of AGNHW. HPLC condition: column, ACE 5 AQ-C18 column (5 μm, 4.6 × 250 mm); mobile phase: gradient profile with acetonitrile (ACN)–0.2% phosphoric acid. 0–10 min, 20% ACN; 10–20 min, 23% ACN; 20–30 min, 25% ACN; 30–35 min, 50% ACN; 35–40 min, 90% ACN. Column temperature: 30°C; flow rate: 1.0 ml/min; injection volume: 10 μl; UV detection: 254 nm.

We first performed HPLC quantitative analysis for quality control of AGNHW. According to the Chinese pharmacopeia (version 2015), baicalin and berberine (**Figure 1A**) were used as standards for *S. baicalensis* Georgi and *C. chinensis* Franch., respectively. In addition, we also included geniposide (**Figure 1A**) as the standard for *G. jasminoides* J. Ellis. **Figure 1B and C** shows the chromatograph for the three standards and AGNHW, respectively. The separation was clear for the three standards. **Table 1** shows the precision, repetition, stability, and recovery test results with HPLC analysis. Our modified HPLC analysis method was stable and reliable for the analysis of AGNHW. The relative standard deviations (RSDs) for the precision of this method fall from 0.64% to 0.88%. The method was also repeatable and stable, with RSD less than 1.74% for repeatability and 1.62% for stability. The average recovery for the three standards was around 98.30% to 99.61%. Meanwhile, the contents of baicalin, berberine, and geniposide were determined as 12.65 ± 0.35, 8.56 ± 0.25, and 13.62 ± 0.47 mg/pill, respectively. These data fell within the requirement of AGNHW as stated in the Chinese pharmacopeia, indicating that the AGNHW we used in this experiment was complied with the national standard.

**Table 1:** Quantitative analysis of AGNHW.

	Precision	Repeatability	Stability	Recovery	Linear regression equation	Correlation coefficient	Contents in AGNHW
Baicalin	0.69%	1.74%	1.60%	99.61%	*y* = 6.821*x* + 28.509	0.9996	12.65 ± 0.35 mg/pill
Berberine	0.64%	0.83%	1.09%	99.74%	*y* = 21.548*x* + 91.648	0.9996	8.56 ± 0.25 mg/pill
Geniposide	0.88%	1.38%	1.62%	98.30%	*y* = 5.6838*x* + 6.7913	0.9995	13.62 ± 0.47 mg/pill

### Angong Niuhuang Wan Ameliorated Neurological Deficits, Reduced Infarct Size, and Preserved Blood–Brain Barrier Integrity

When choosing the dosage of AGNHW, we had performed a preliminary experiment by using 128.5 mg/kg (equivalent to 0.5× AGNHW), 257 mg/kg (equivalent to 1× AGNHW), and 514 mg/kg (equivalent to 2× AGNHW). Results showed that 257 and 514 mg/kg AGNHW could significantly protect rats against cerebral ischemia–reperfusion injury. There was no significant difference in the neuroprotection between the doses of 257 and 514 mg/kg AGNHW (**Supplementary Figure 1**). Since 257 mg/kg is the clinical dose, we selected this dose to proceed to the following experiments.

**Figure 2:**
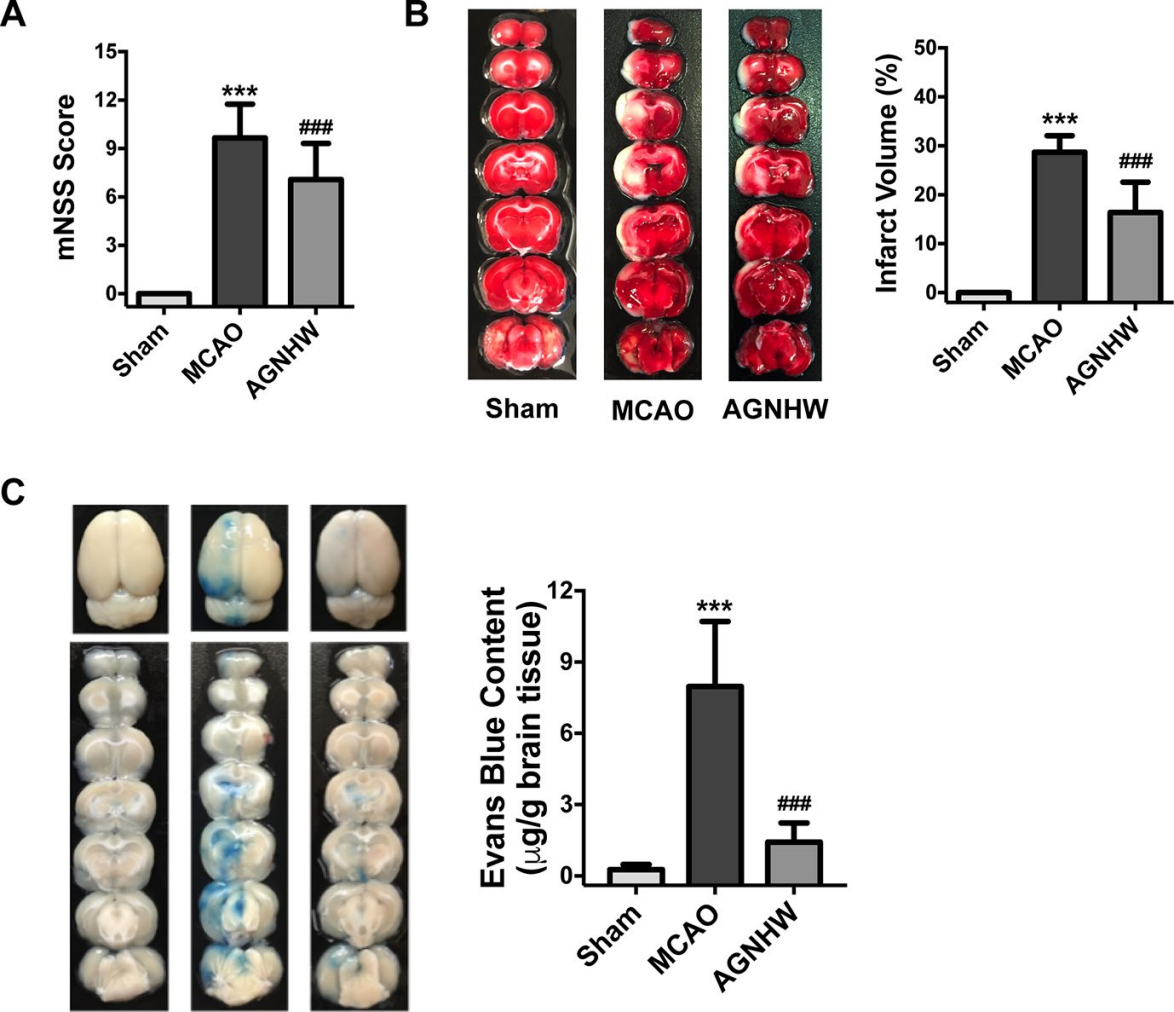
AGNHW ameliorated middle cerebral artery occlusion (MCAO)-induced neurological deficits, decreased infarct size, and preserved blood–brain barrier (BBB) integrity. Male Sprague–Dawley (SD) rats were subjected to 2 h of ischemia and 22 h of reperfusion to establish the MCAO model. AGNHW (257 mg/kg, suspended in saline) was given to animals before reperfusion. **(A)** Modified Neurological Severity Score (mNSS) was evaluated 24 h after the onset of ischemia–reperfusion based on an 18-point scale (*n* = 12). **(B)** Infarct size measured by TTC staining 24 h after the onset of ischemia–reperfusion (*n* = 10). **(C)** Evans blue content measured 24 h after the onset of ischemia–reperfusion (*n* = 10). All data are means ± S.E.M. The significance of differences was from Sham at ***p < 0.001 and from MCAO at ^###^ *p* < 0.001.

Neurological deficit was evaluated at 24 h after the onset of ischemia with an 18-point scale. The higher the point awarded to the animal, the more neurological deficit scores were observed. Compared with the MCAO group, the neurological deficit score was markedly reduced in the AGNHW (257 mg/kg)-treated group (**Figure 2A**). The recovery of neurological functions is one of the most important factors in the clinical treatment of stroke. This result suggests that AGNHW could have a prominent neuroprotective effect on the animals with cerebral ischemia–reperfusion injury. Therefore, we further examined the infarct size and the BBB integrity in the ischemic brains. Results showed that the infarct portion in both striatum and lateral cortex of the left hemisphere was significantly reduced after oral administration of AGNHW (**Figure 2B**), and the extensive extravasation of Evans blue dye in the left hemisphere was also decreased by AGNHW treatment (**Figure 2C**). These results suggested that AGNHW had neuroprotective effects against cerebral ischemia–reperfusion injury. AGNHW could reduce the infarct size and protect the BBB integrity in cerebral ischemia–reperfusion injury.

### Angong Niuhuang Wan Protected Ischemic Brains by Attenuating Excess Apoptosis, Oxidative and Nitrative Stress, and Protected Tight Junction Proteins

**Figure 3:**
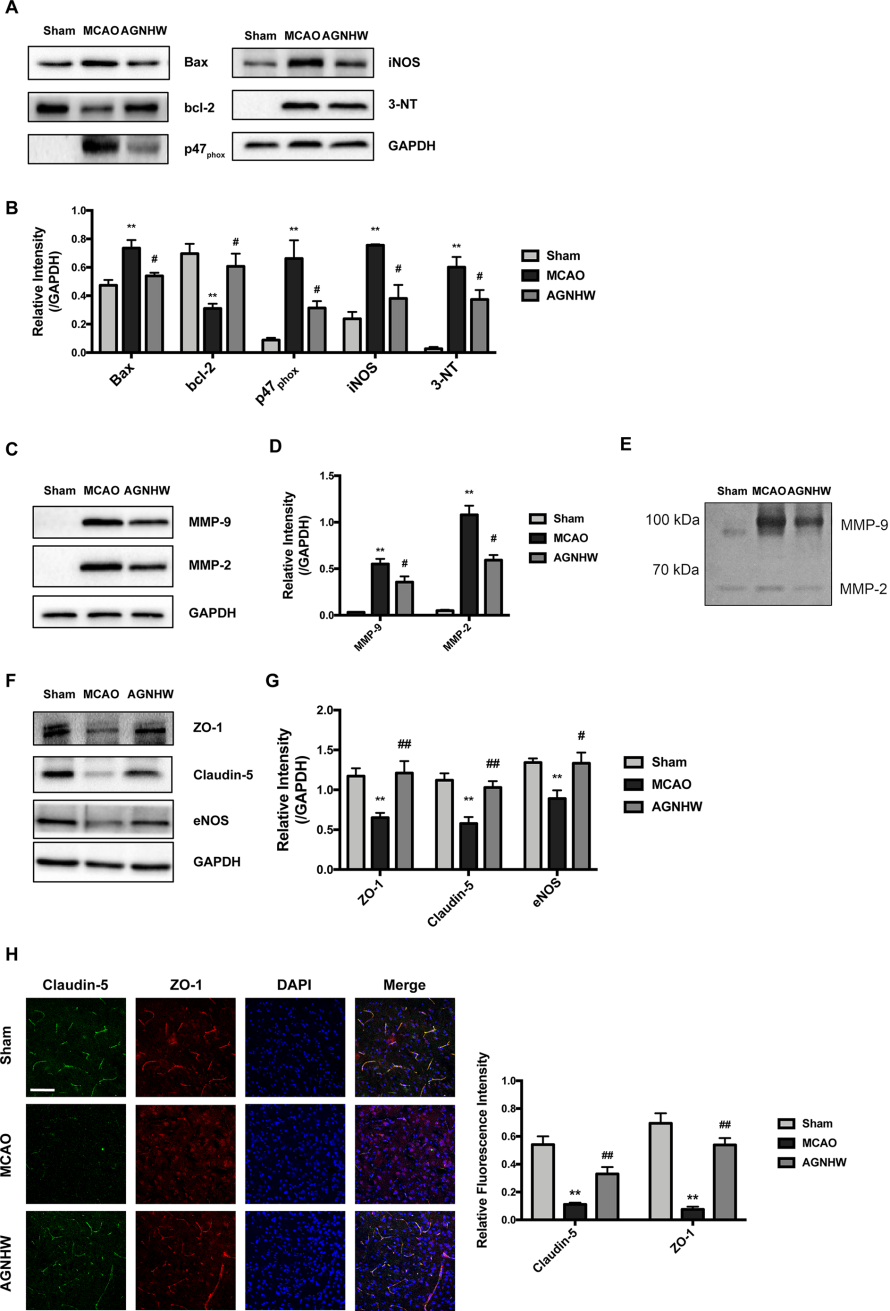
AGNHW protected both brain tissues and microvessels in animals suffering from ischemia-reperfusion injury. Brain tissues and microvessels were obtained from ipsilateral sides of the brain of MCAO rats subjected to 2 h of ischemia and 22 h of reperfusion for Western blot analysis, gelatin zymography, and immunofluorescence study. **(A** and **B)** Protein expressions of apoptotic pathway, superoxide production, nitric oxide production, and nitration end-products in the brain. **(C** and **D)** Matrix metalloproteinase (MMP) expression in microvessels. **(E)** Gelatin zymography showing MMP activities in microvessels. **(F** and **G)** TJ protein expression in microvessels. **(H)** Immunofluorescence images on TJ proteins located in the cerebral cortex. Scale bar = 100 μm. All data are means ± S.E.M. The significance of differences was from Sham at **p < 0.01 and from MCAO at ^#^ *p* < 0.05, ^##^ *p* < 0.01.

We then analyzed the protein expressions of the ipsilateral sides of the brains to investigate its possible pharmacological mechanisms. Results showed that MCAO ischemia–reperfusion induced significant increases in the expression of Bax but reduced bcl-2 in the ipsilateral sides of the rat brains (**Figure 3A and B**). The expression of p47_phox_ (NADPH oxidase subunit) and nitric oxide synthase (iNOS) were significantly increased in the MCAO ischemia-reperfused brains. Meanwhile, the expression of 3-NT (ONOO^−^ footprint) was also significantly increased. Oral administration of AGNHW up-regulated Bcl-2 expression and down-regulated the expression of Bax, p47_phox_, iNOS, and 3-NT in the ischemic brains (**Figure 3A and B**). These results suggest that AGNHW has antioxidant properties against oxidative and nitrative stress in cerebral ischemia–reperfusion injury.

To assess the protective effects of AGNHW on the BBB integrity in the cerebral ischemia–reperfusion injury, we extracted the microvessels in the ipsilateral sides of the ischemic brains and examined the expression of MMP-9, MMP-2, and TJ proteins including ZO-1 and claudin-5. As shown in **Figure 3C–E**, MCAO ischemia–reperfusion induced the expression and activity of MMP-9 and MMP-2 as showed by Western blot and gelatin zymography analysis, respectively. AGNHW treatment remarkably down-regulated the expression and activities of MMP-2 and MMP-9. MCAO ischemia–reperfusion significantly down-regulated the expression of ZO-1 and claudin-5, and AGNHW treatment reserved the expression of ZO-1 and claudin-5 in the ischemic brains, preventing the loss of TJ protein expression in the ischemic brains (**Figure 3F and G**). Consistently, the expression of eNOS in microvessels was also recovered after AGNHW administration (**Figure 3F and G**). Furthermore, we detected the expression of claudin-5 and ZO-1 in brain slices using confocal microscopy. Immunofluorescent images showed a sparse expression of claudin-5 and ZO-1 in the ischemic rat brains after MCAO ischemia–reperfusion. AGNHW administration significantly increased the expression of claudin-5 and ZO-1 (**Figure 3H**). These results implied that treatment of AGNHW could inhibit the expression and activity of MMP-2 and MMP-9, and prevented the degradation of ZO-1 and claudin-5 in the ischemic brains in cerebral ischemia–reperfusion injury.

### Angong Niuhuang Wan Did Not Exhibit Significant Liver and Kidney Toxicity in Normal Rats with Overdose Treatment

**Figure 4:**
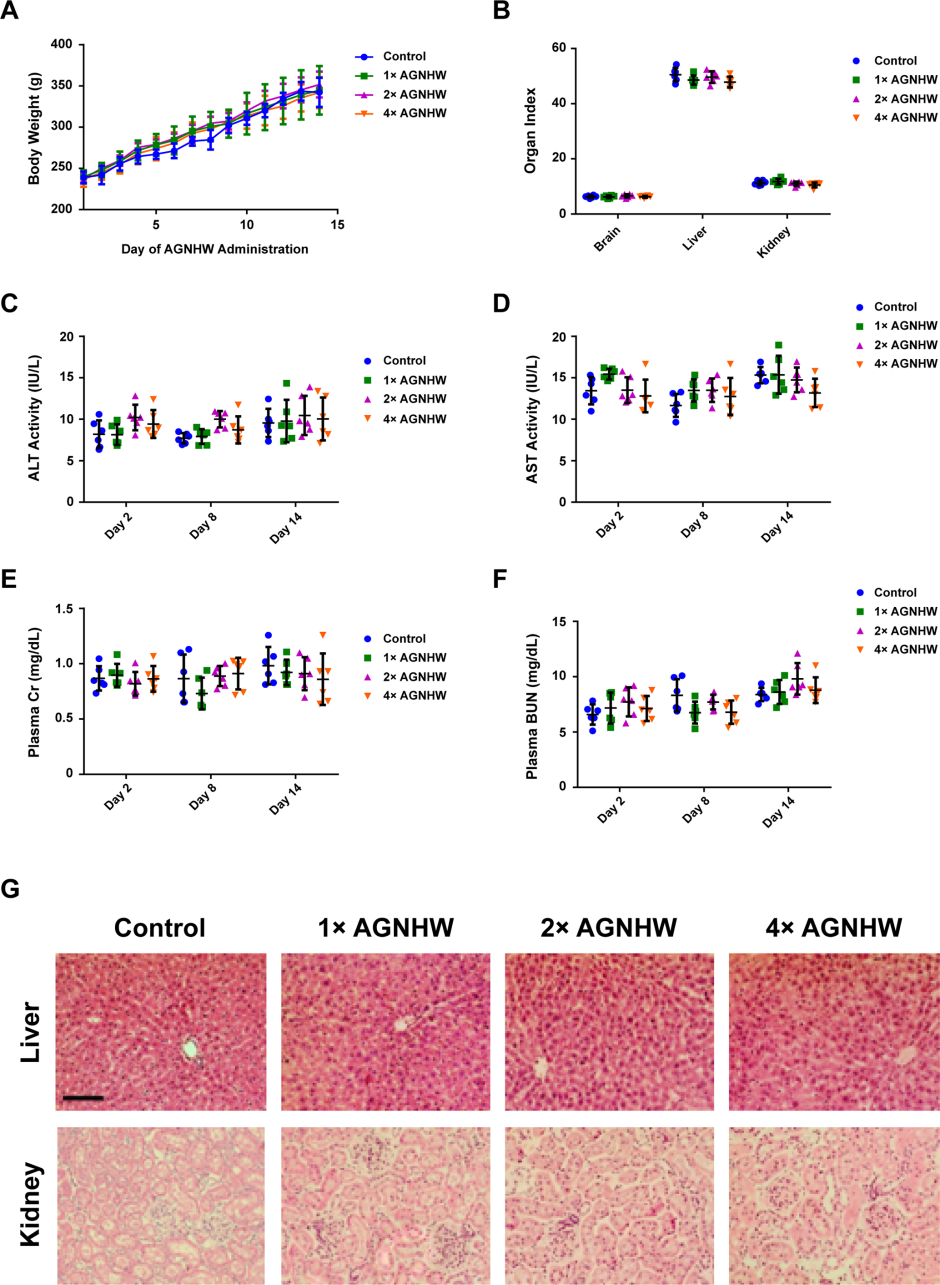
AGNHW did not influence liver and kidney functions in normal rats. AGNHW was given to normal rats at three concentrations (257, 514, and 1,028 mg/kg) for seven consecutive days. Blood samples were collected on day 2, day 8, and day 14 after the first administration. Organ samples were collected on day 14 after the first administration. **(A)** Body weight changes. **(B)** Organ index. **(C)** ALT and **(D)** AST activity in the plasma. **(E)** Creatinine and **(F)** BUN level in the plasma. **(G)** Histological examination on liver and kidney morphology. Scale bar = 100 μm.

Although AGNHW has a potent neuroprotective effect in protecting ischemia–reperfusion injury, its heavy metal content (realgar and cinnabar) still raise concerns on the safety of using this famous traditional prescription. Therefore, we evaluated the toxicology of consuming a larger dose of AGNHW for seven consecutive days in normal animals. All animals were sacrificed 7 days after the last oral administration. As shown in **Figure 4A and B**, a continuous large dose of AGNHW up to 1,028 mg/kg (4× AGNHW) did not cause any alteration in the body weight of the rats. It also did not cause changes in organ index. We then determined the liver and kidney functions in these animals. **Figure 4C–F** shows that the changes in plasma ALT, AST, Cr, and BUN of the rats administered with 4× AGNHW whose changes were not significant when compared to control animals at all three time points. Further evidence was shown in **Figure 4G**, where H&E staining of liver and kidney showed no abnormal morphology. These results indicate that there should be no potential toxicity when AGHWH is used within a week.

### Heavy Metal Level in the Blood and Organs After Angong Niuhuang Wan Administration

**Figure 5:**
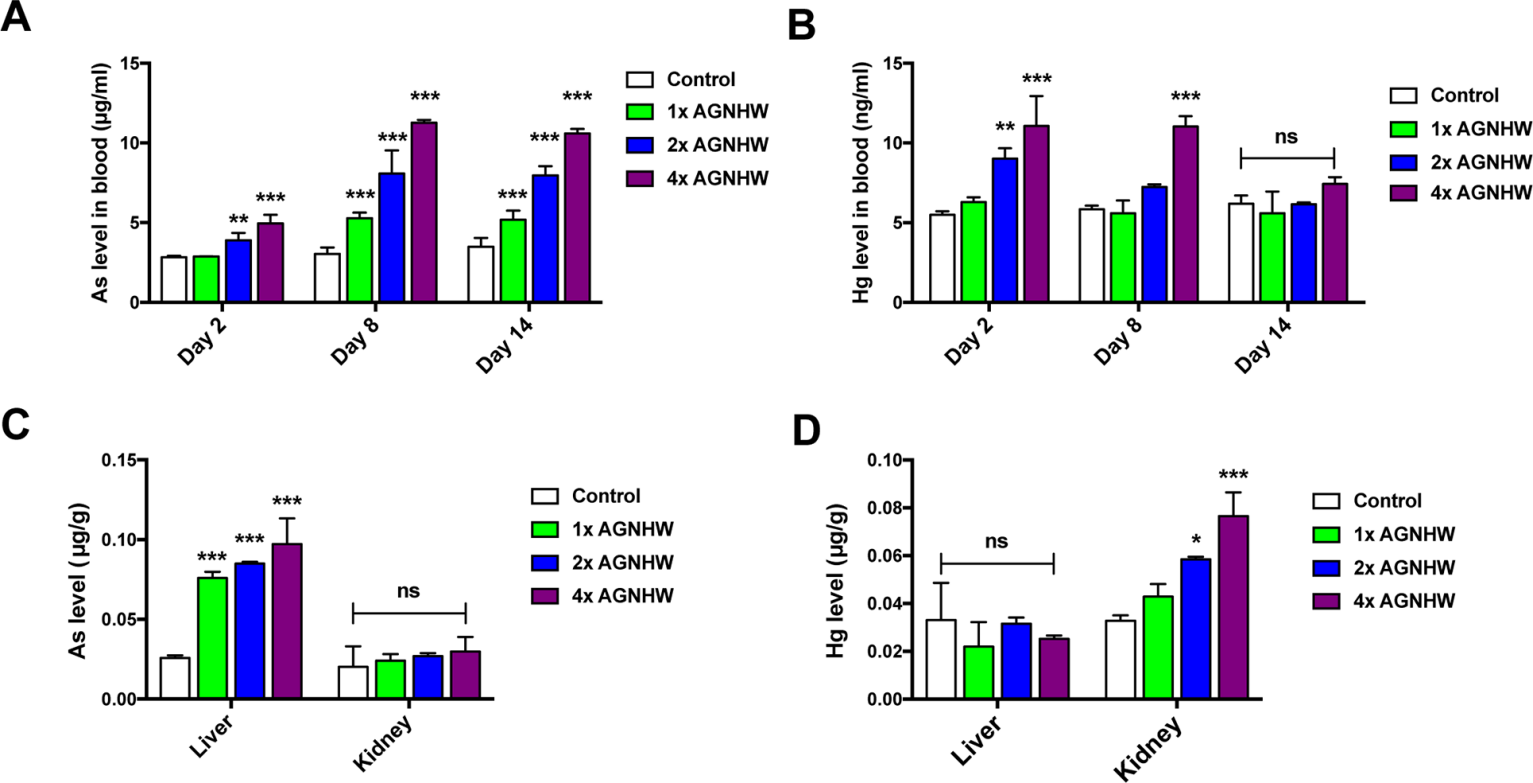
Heavy metal profile after AGNHW continuous administration in normal rats. AGNHW was given to normal rats at three concentrations (257, 514, and 1,028 mg/kg) for seven consecutive days. Blood samples were collected on day 2, day 8, and day 14 after the first administration. Organ samples were collected on day 14 after the first administration. All samples were acid digested with concentrated nitric acid and oxidized with hydrogen peroxide. All data were analyzed using inductive coupled plasma–mass spectrometry (ICP-MS). **(A)** Blood arsenic level and **(B)** mercury level on day 2, day 8, and day 14 after the first administration. **(C)** Organ arsenic level and **(D)** mercury level on day 14 after the first administration. All data are means ± S.E.M. The significance of differences was from control at *p < 0.05; **p < 0.01; ***p < 0.001. No significance was marked as ns.

In order to gain a deeper understanding on the heavy metal profile after oral administration of AGNHW, we collected blood samples from the rats at three time points. The organ samples were collected at the end of the experiment for the determination of heavy metal content using ICP-MS. **Figure 5A and B** was the arsenic and mercury level in blood at different time points. The consumption of 1× AGNHW for 7 days had no significant change in the level of mercury in the blood during the experiment. However, continuous consumption of AGNHW for 7 days led to a nearly doubled increase of arsenic in the blood at day 8. This level of arsenic was not eliminated till day 14; even AGNHW administration had stopped for 7 days. It is understandable that the consumption of 2× AGNHW and 4× AGNHW significantly increased the levels of arsenic and mercury in the blood. Although there were high levels of mercury found in the blood in the rats treated with 2× and 4× AGNHW for 7 days, the mercury level was eliminated and restored nearly back to normal at day 14 afterwards. 

Apart from arsenic and mercury levels in the blood, we also detected those heavy metal levels in the liver and kidney of the rats. As shown in **Figure 5C and D**, we noticed that arsenic was mainly accumulated in the liver, while mercury was found in the kidney. After administration of AGNHW for 7 days, the level of arsenic in the liver tissues was significantly increased when compared to the control group. The arsenic concentration was further increased when the doses of 2× AGNHW and 4× AGNHW were administered to the rats. Interestingly, there was no significant increase in mercury level in the kidney after the rats were orally administered with 1× AGNHW. Similar to arsenic level in the liver, the renal mercury level was increased when the rats was administered with 2× and 4× AGNHW. Thus, although AGNHW may increase the arsenic in the liver and induce the accumulation of mercury in the kidney, no renal and liver function would be affected even the rats were treated with high doses of AGNHW (2× and 4× AGNHW). Therefore, short-term treatment of AGNHW within 1 week should be relatively safe as the formula is generally used as the first-aid treatment for acute ischemic stroke in TCM practice.

## Discussion

AGNHW is one of the most valuable first-aid drugs for stroke treatment in TCM with a long history in China and Southeast Asia (Pan et al., 2001). There are numerous clinical reports on the neuroprotective effects of AGNHW in ischemic stroke patients (Guo et al., 2014). However, seldom well-designed toxicological studies at both basic science and clinical trial can be found in literature. The non-standardized clinical trial results could not provide sufficient support for proving the efficacy of AGNHW in stroke treatment. Meanwhile, the heavy metal elements, i.e., realgar and cinnabar, creates major concerns on the safety of AGNHW. With these heavy metal components, AGNHW is not allowed to be sold in the US and European markets (Ernst, 2002). Lu et al. (2011a, 2011b, 2011c) reported that AGNHW was less toxic than the commonly used arsenicals and mercurials. Realgar and cinnabar are in the inert form of the metal element, making them less toxic (Wu et al., 2011). However, Wang et al. (2015) reported that sub-chronic use of cinnabar could cause renal injury. Low-dose realgar-containing herbal products may induce mild kidney injury after long-term use (Luo et al., 2017). Therefore, it is desirable to have well-designed experiments to systematically study the neuroprotective effects and toxicological effects for guiding uses of the patients.

In the present study, we obtained the following experimental results and implications: 1) AGNHW reduced the infarct size and protected the BBB integrity in the MCAO rat ischemic stroke model. The underlying neuroprotective mechanisms of AGNHW could be associated with its antioxidant properties by inhibiting oxidative/nitrative stress-mediated MMP activation and protecting TJ proteins in the ischemic brains; 2) Administration of 1×AGNHW for 7 days would not induce the accumulation of mercury in blood, liver, and kidney at day 14. Administration of 2×AGNHW and 4×AGNHW for 7 days would not induce accumulation of mercury in blood and liver but increase the level of mercury in the kidney at day 14. For arsenic level, administration of 1×AGNHW for 7 days would increase the level of arsenic in the liver and blood without increasing arsenic in the kidney at day 14. Administration of 2×AGNHW and 4×AGNHW for 7 days would further increase the level of arsenic in the liver and blood. However, there were no significant differences in body weight, organ index, histological structures, and renal and liver functions even the rats were treated with high dose of 4× AGNHW with the increased level of arsenic in the liver and mercury in the kidney. Our results suggest that short-term treatment of 1× AGNHW within 1 week should be safe and has neuroprotective effects against cerebral ischemia–reperfusion injury.

It is well known that cerebral ischemia–reperfusion could produce a large amount of ROS/RNS to induce oxidative/nitrative stress and MMP activations, leading to BBB disruption (Chen et al., 2013; Chen et al., 2018). Our previous studies demonstrated that RNS, especially peroxynitrite, plays crucial roles in activating MMPs, destroying the BBB and inducing neurological deficits in cerebral ischemia–reperfusion injury (Chen et al., 2013;Chen et al., 2015; Feng et al., 2018a). Targeting ROS/RNS-mediated MMP activation could be a crucial therapeutic strategy for protecting the BBB integrity and improving the therapeutic outcome in the treatment of ischemic stroke. In the present study, oral administration of 1× AGNHW, equivalent to the human dose, significantly protected the MCAO rat from cerebral ischemia–reperfusion injury. AGNHW treatment up-regulated Bcl-2 expression and down-regulated the expression of Bax, p47_phox_, iNOS, and 3-NT in the ischemic brains. These results indicate that AGNHW had inhibitory effects on oxidative and nitrative stress in cerebral ischemia–reperfusion injury. The antioxidant properties of AGNHW could be contributors to its neuroprotective effects against cerebral ischemia–reperfusion injury.

TJs are important components in the BBB. MMP activation can mediate TJ protein degradation, as they are the major enzyme systems involved in the extracellular matrix. The presentation of TJ protein is crucial to the maintenance of BBB integrity (Sandoval and Witt, 2008). We found that AGNHW administration inhibited MMP activity in the microvessels of the ischemic brains and reserved the expression of ZO-1 and claudin-5, two representative TJ proteins. Our recent study indicate that ONOO^−^-mediated MMP activation plays crucial roles in BBB disruption, HT, and poor outcome in ischemic stroke rats with delayed thrombolytic treatment (Zhang et al., 2012; Chen et al., 2018). AGNHW could inhibit the ONOO^−^-mediated MMP activation and preserve the BBB integrity in cerebral ischemia–reperfusion injury. Thus, AGNHW could be a potential adjunct therapy with t-PA to reduce the BBB disruption and HT, and increase the outcome for ischemic stroke patients with delayed t-PA treatment. This needs to be further investigated in the future.

According to the manufacturer, AGNHW should not be consumed for more than three consecutive days. Therefore, we tested whether consuming a larger dose of AGNHW for a longer period of time would have an adverse effect on the animal. As arsenic and mercury mainly affect liver and kidney (Tinggi et al., 2016), we performed the experiments for testing liver and renal function in the rats that consumed different dosages of AGNHW for 7 days and allowed the animals to recover for 7 days before sample collection. We found that a large dose of AGNHW, even quadruple level of normal dose, would not significantly influence body weight, organ index, liver functions, kidney functions, and organ morphology. This might be due to the sulfide forms of realgar and cinnabar, which were suggested to be inert even in the human body (Wu et al., 2011;Zhou et al., 2011). However, our results also revealed that arsenic and mercury were indeed accumulated in the rats treated with double and quadruple doses of AGNHW. We found that arsenic elimination was not as efficient as mercury. The level of arsenic in the blood was doubled in the 1× AGNHW group and tripled in the 4× AGNHW group. Arsenic level in liver was also tripled in the rats treated with 1× AGNHW treatment. It is interesting to find that mercury level was rather stable in all detection time points for both blood and organs. According to these data, we could understand that taking a high dose of AGNHW for a longer time was probably the reason for adverse reaction and toxicity. However, if AGNHW was consumed with the correct dose and duration, it is definitely beneficial to the treatment of ischemic stroke. Long-term consumption of AGNHW is not recommended.

In conclusion, AGNHW could decrease infarct size and protect the BBB integrity during cerebral ischemia–reperfusion injury. The underlying mechanisms of AGNHW could be attributed to its antioxidant properties by inhibiting oxidative/nitrative stress-mediated MMP activation and protecting TJ proteins. Although AGNHW consists of arsenic- and mercury-containing mineral materials, the use of AGNHW at a regular dose within 1 week should be at low possibility to induce the accumulations of arsenic and mercury and influence liver and kidney functions. Therefore, we conclude that AGNHW is relatively safe when used as a first-aid treatment approach for acute ischemic stroke.

## Ethics Statement

This study was carried out in accordance with the recommendations of Guidelines for the Use of Experimental Animals, the Committee on the Use of Live Animal in Teaching and Research, The University of Hong Kong. The protocol was approved by the Committee on the Use of Live Animal in Teaching and Research, The University of Hong Kong.

## Author Contributions

BT and JS designed the outline of the study and prepared the final manuscript. BT performed the experiments, acquisition, analysis of data, and statistical analysis. XC helped to facilitate the animal experiments. CG contributed to the immunofluorescence studies. SW, SY, and DY were involved in the HPLC study and the ICP-MS study. JS received funding and guided all of the studies. All authors read and approved the final manuscript.

## Funding

This study was supported by the Hong Kong Innovation and Technology Commission ITF grant (UIM/289); the ITF Internship Programme (InP/246/17); Hong Kong General Research Fund (GRF No. 17118717), Research Grant Council, Hong Kong SAR, China; Grant from National Natural Science Foundation of China (No. 31570855) and Areas of Excellence Scheme (AoE/P-705/16), Research Grant Council, Hong Kong SAR, China.

## Conflict of Interest Statement

The authors declare that the research was conducted in the absence of any commercial or financial relationships that could be construed as a potential conflict of interest.
