# Supplementary material for: Neuroprotective Effects and Hepatorenal Toxicity of Angong Niuhuang Wan Against Ischemia–Reperfusion Brain Injury in Rats
Source: Front Pharmacol. 2019 May 29;10:593. doi: 10.3389/fphar.2019.00593 (PMC6548905; doi:10.3389/fphar.2019.00593)
Supplement: Supplementary file 1 [file Image_1.pdf]

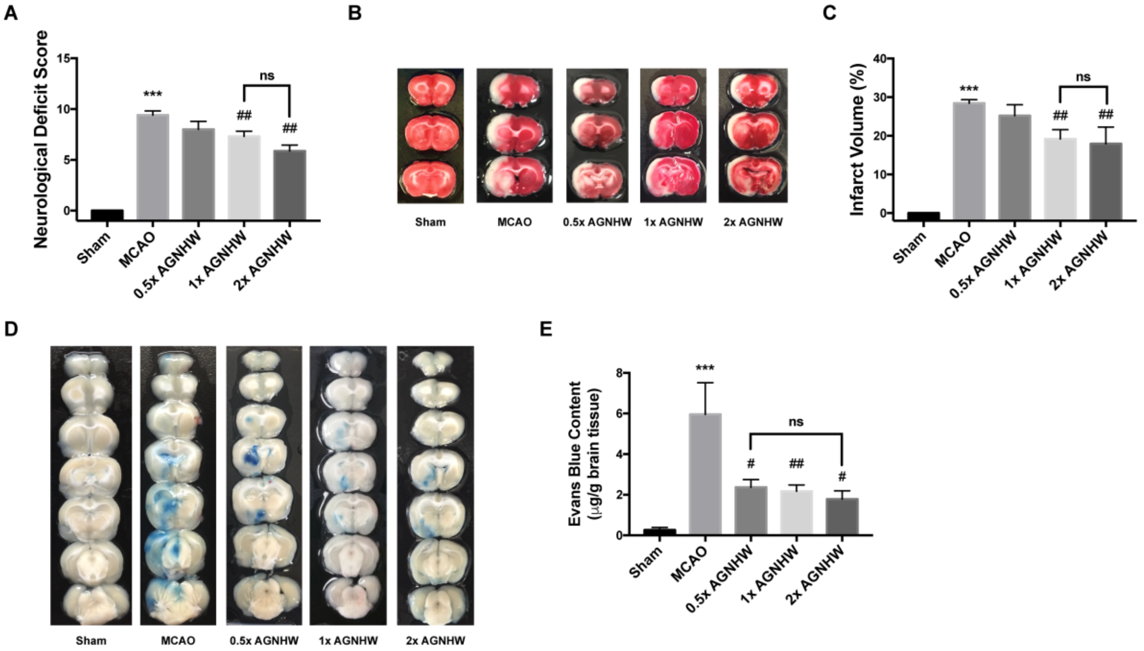

Supp. Fig. 1 AGNHW ameliorated MCAO-induced neurological deficits, decreased infarct size and preserved BBB integrity.

Male SD rats were subjected to 2 h of ischemia and 22 h of reperfusion to establish MCAO model. AGNHW (128.5, 257 and 514 mg/kg, suspended in saline) was given to animals before reperfusion. (A) Modified Neurological Severity Score (mNSS) was evaluated on 24 h after the onset of ischemia-reperfusion based on an 18-point scale (n = 8). (B) Infarct size measured by TTC staining on 24 h after the onset of ischemia-reperfusion (n = 8). (C) Evans blue content measured on 24 h after the onset of ischemia-reperfusion (n = 8). All data are means ± S.E.M. The significance of differences was from Sham at $^{***}p < 0.001$; from MCAO at $^{\#}p < 0.05$; $^{\#\#}p < 0.01$. No significance was marked as ns.
